# Knockout mutation in *TaD27* enhances number of productive tillers in hexaploid wheat

**DOI:** 10.3389/fgeed.2024.1455761

**Published:** 2024-10-14

**Authors:** Muhammad Jawad Akbar Awan, Imran Amin, Awais Rasheed, Nasir A. Saeed, Shahid Mansoor

**Affiliations:** ^1^ Agricultural Biotechnology Division, National Institute for Biotechnology and Genetic Engineering (NIBGE), Constituent College Pakistan Institute of Engineering and Applied Sciences (PIEAS), Faisalabad, Pakistan; ^2^ Department of Plant Sciences, Quaid-i-Azam University, Islamabad, Pakistan; ^3^ State Key Laboratory of Crop Gene Resources and Breeding/National Engineering Research Center of Crop Molecular Breeding, Institute of Crop Sciences, Chinese Academy of Agricultural Sciences (CAAS), Beijing, China; ^4^ Jamil ur Rehman Center for Genome Research, International Center for Chemical and Biological Sciences, University of Karachi, Karachi, Pakistan

**Keywords:** genome editing, DWARF27, strigolactones, wheat yield, negative regulator, wheat mature embryos

## Abstract

Recent advances allow the deployment of cluster regularly interspaced short palindromic repeats (CRISPR)-associated endonucleases (Cas) system for the targeted mutagenesis in the genome with accuracy and precision for trait improvement in crops. CRISPR-Cas systems have been extensively utilized to induce knockout or frameshift mutations in the targeted sequence of mostly negative regulating genes for wheat improvement. However, most of the reported work has been done in non-commercial varieties of wheat and introgression of edited alleles into breeding population comes with the penalty of unwanted linkage-drag. Wheat yield is controlled by various genes such as positive and negative regulators. The *TaD27* gene is described as a negative regulator of shoot branching or tillering and involved in the biosynthesis of strigolactones. In this study, we developed *Tad27* knockout mutant lines of an elite wheat cultivar that showed a twofold increase in the number of tillers and 1.8-fold increase in the number of grains per plant. Subsequently, enhancing the grain yield without any morphological penalty in the architecture of the plants. The co-transformation of regeneration enhancing growth regulator, G*rowth Regulating Factor 4* (*GRF4*) and its cofactor *GRF-Interacting Factor 1* (*GIF1*), under single T-DNA cassette improved the regeneration efficiency up to 6% of transgenic events from mature embryos of wheat. Our results indicate that the CRISPR-mediated targeted mutagenesis confers the potential to knockout yield-related negative regulators in elite cultivars of wheat that can substantially enhance grain yield per plant and this strategy can be harnessed for the improvement of future wheat.

## 1 Introduction

With a growing population, global food security demands rapid and sustainable measures to cope with the foreseen threat of hunger. Hexaploid wheat (*Triticum aestivum* L.) is a staple crop for a large population around the globe and a major source of nutrition and calories, providing essential ingredients such as protein, carbohydrates, and fibres (Awan et al., 2022). To improve the wheat yield, various traditional techniques have been utilized such as conventional breeding to generate phenotypically better-performing germplasm by crossing discrete cultivars or induction of random mutagenesis through physical or chemical treatments (Uauy et al., 2017). However, these practices require labour-intensive selection and screening protocols. In contrast, marker-assisted selection (MAS) and target-induced local lesions in the genome (TILLING) have fast-tracked the development of novel germplasm (Uauy et al., 2017; Rasheed and Xia, 2019). Recent advances allow the deployment of engineered nucleases such as zinc finger nucleases (ZFNs), transcription-activator like effector nucleases (TALENs), and cluster regularly interspaced short palindromic repeats (CRISPR)- and associated (Cas) endonucleases for the targeted mutagenesis in the genome with accuracy and precision (Zhu et al., 2020). Among these genome editing systems, CRISPR-Cas9 has been widely employed in wheat for various agronomically significant traits such as grain yield enhancement, quality improvement, and biotic/abiotic resistance (Awan et al., 2022).

CRISPR-Cas systems have been extensively used to induce knockout or frameshift mutations in the targeted sequence of mostly negative regulating genes (Zhu et al., 2020). Recently, the CRISPR-Cas9 system has been utilized to delete a large chunk of DNA (304 kb) in wheat, harbouring a susceptibility gene, that conferred resistance against powdery mildew disease without imposing any yield penalty (Li et al., 2022). Similarly, this system has been employed to improve plant architecture such as the number of seeds per spike and thousand-grain weight (TGW) by knocking out the *TaCKX2.1* and the *TaGW2* genes, respectively (Shan et al., 2014; Zhang et al., 2019; Lin et al., 2020). The *TaGW2* gene is a negative regulator of grain weight that encodes RING-type protein, an E3 ubiquitin ligase, and is involved in the cellular degradation of targeted proteins (Song et al., 2007). The *TaCKX2.1* gene is also a negative regulator of grain yield and encodes cytokinin oxidase/dehydrogenase enzyme that is involved in the degradation of cytokinin, an essential plant growth hormone (Jameson and Song, 2015). A targeted mutation in the *abnormal cytokinin response1 receptor 1* (*are1*) gene delayed senescence and improved nitrogen use efficiency and grain yield in wheat (Zhang et al., 2021).

The *DWARF27* (*D27*) gene encodes beta-carotenoid isomerase and is reported to be involved in controlling shoot branching (tillering) in rice and wheat (Lin et al., 2009; Zhao et al., 2020). The beta-carotenoid isomerase initiates a cascade of reactions that results in the biosynthesis of strigolactones (SLs), the carotenoid-driven hormones that control plant growth and development and act as the inhibitors of axillary bud outgrowth (Supplementary Figure S1). The D27 isomerase converts all-*trans*-*beta*-carotene to *9*-*cis-beta*-carotene that acts as substrate for CAROENOID CLEAVAGE DIOXYGENASE7 (CCD7) and CAROENOID CLEAVAGE DIOXYGENASE8 (CCD8) enzymes to yield carlactone, a precursor for SLs biosynthesis (Waters et al., 2017). Previously, the *d27* mutants showed reduced SLs and enhanced outgrowth of axillary buds in *Arabidopsis* and rice (Lin et al., 2009; Waters et al., 2012). In wheat, the *TaD27*-RNAi plants showed increased shoot branching with no signs of defective growth or yield penalties in transgenic plants, in contrast, the *TaD27-B*-overexpressing line demonstrated a reduced number of tillers (Zhao et al., 2020).

Tillering is a major agronomic trait that can play a positive role in yield enhancement in wheat. CRISPR-Cas9-mediated genome editing offers transgene-free modifications in the genome in a target-specific manner (Zhu et al., 2020). Moreover, a recent study reported that the co-transformation of G*rowth Regulating Factor 4* (*GRF4*) and its cofactor *GRF-Interacting Factor 1* (*GIF1*) can increase regeneration efficiency in wheat calli (Debernardi et al., 2020). It was hypothesized that the knockout mutants of the *TaD27* gene can promote shoot branching and the number of seeds per plant, eventually, an enhanced grain yield can be achieved. In the present study, two knockout mutants of *TaD27* genes were generated by deploying CRISPR-Cas9 machinery along with a regeneration-enhancing *GRF4*-*GIF1* cassette through *Agrobacterium*-mediated transformation in a commercial cultivar. Our results demonstrated that the *Tad27* mutants showed an increased number of tillers and the number of grains per plant as compared to wildtype plants.

## 2 Materials and methods

### 2.1 Plant materials

Three months old post-harvested seeds of a commercially cultivated elite wheat variety GA-2002 (GA) were utilized for the isolation of mature embryos. The mature seeds were collected in a 50 mL falcon tube and rinsed with autoclaved double distilled water (ddH_2_O). A 60-s rapid wash was given with 70% ethanol (v/v) for surface sterilization, followed by a quick rinse with ddH_2_O in a laminar airflow cabinet. The seeds were placed in a 0.1% solution of mercuric chloride (w/v) aided with 1-2 drops of Tween-20, on a shaker with 80 rotation per minute (rpm) speed for 7–10 min. Then the seeds were washed three times with ddH_2_O. The surface sterilized mature seeds were imbibed in 5 mg L^−1^ solution of 2,4-Dichlorophenoxyacetic acid (2,4-D) (PhytoTech Labs, United States) (w/v) at 28°C shaker in the dark overnight.

### 2.2 Binary construct development and *Agrobacterium* transformation

To develop a pJ32 binary vector for wheat transformation, the pRGEB32 plasmid (Addgene plasmid #63142; Xie et al., 2015) was modified by replacing the *OsU3* promoter with *Ta*U6.3 promoter by using *Hind*III and *Bsa*I (New England Biolabs, MA, United States) restriction enzymes to generate pRGEB32-TaU6.3 vector. The *TaU6.3* promoter was amplified from the wheat genome by using TaU6.3-Hind-F 5′-TAC​GCC​AAG​CTT​ACT​CTG​CGA​GGA​CTT​CTC​TTG​TGA​AAA​G3′ and TaU6.3-Bbs-R 5′-AAA​TGT​GGA​AGA​CCC​AAA​CCG​AGA​CCG​GAT​CCG​GTC​TCC​GAG​TCT​GTG​TGC​AGC​GCG​TGG​C3′ primers and cloned into restricted pRGEB32 after restriction digestion with *Hind*III and *Bbs*I enzymes (New England Biolabs, MA, United States). Furthermore, the pJD553 (Addgene plasmid #160392; Debernardi et al., 2020) was used as a template to amplify the *Zm*UbiPro:*GRF4*-*GIF1*:NosT cassette by using GRF-Hind-F 5′-AAT​TTA​AGC​TTT​AAT​AAA​TAG​ACA​CCC​CCT​C3′ and GRF-Hind-R 5′-AAT​TTA​AGC​TTC​TGC​AGG​CGC​GCT​AAT​CCC​C3′ primers. All the polymerase chain reactions for cloning were performed by using Platinum™ SuperFi II DNA Polymerase (Thermo Fisher Scientific, MA, United States). The amplified *Zm*Ubi: *GRF4*-*GIF1*: NOS cassette was cloned into the pRGEB32-TaU6.3 vector by using *Hind*III (New England Biolabs, MA, United States) enzyme. The final binary construct, termed pJ32, contained a *Bsa*I site for the cloning of single guide RNA (sgRNA) under *TaU6.3* promoter, *Os*UbiPro:*Os*Cas9:NosT cassette, *Zm*UbiPro:*GRF4*-*GIF1*:NosT cassette, and CaMV35Pro*Hpt*:CaMVT cassette (hygromycin resistance gene-selectable marker) in T-DNA cassette. The guide RNAs (gRNAs) having 20-nucleotides (nt) long complementary oligos, bearing target site sequences against *TaD27* gene, were designed manually by aligning homeologs sequences and assessed for off-targets effect with up to three-mismatches by using Cas-OFFinder online tool (www.rgenome.net/cas-offinder/). The oligos were synthesized with extra four nucleotides as overhangs on 5′- ends such as 5′-ACTC and 5′-AAAC. After T4 Polynucleotide Kinase (PNK) treatment (New England Biolabs, MA, United States), the self-annealed oligos were cloned into pJ32 vector between *TaU6.3* promoter and scaffold by using *Bsa*I restriction enzyme (Supplementary Figure S2). Two constructs were generated each carrying a different gRNA for targeted mutagenesis. The final constructs were confirmed through Sanger sequencing. The verified constructs were transformed into hypervirulent *Agrobacterium tumefacien* strain EHA105. The pRGEB32 plasmid without *GRF4*-*GIF1* cassette was used as control to assess the regeneration efficiency of pJ32-carrying *GRF4*-*GIF1* cassette.

### 2.3 Wheat mature embryos isolation

The overnight imbibed seeds were removed from a 5 mg/mL solution of 2,4-D in a Petri dish within a laminar flow hood under aseptic conditions. The mature embryos were isolated with the aid of fine forceps and scalpel. The isolated embryos were placed into 1.5 mL centrifuge tube carrying 1 mL wheat inoculation media (WIM), prepared by following the recipe provided by (Hayta et al., 2021), containing 0.44 mg L^−1^ Murashige and Skoog Basal Medium with Vitamins (MS) (PhytoTech Labs, KS, United States), 10 g L^−1^ glucose (PhytoTech Labs, KS, United States), 0.5 g L^−1^ 2-(N-morpholino) ethanesulfonic acid (MES) (PhytoTech Labs, KS, United States), 0.05% Silwet L-77 (PhytoTech Labs, KS, United States) and freshly added 100 µM acetosyringone (PhytoTech Labs, KS, United States) before use. The isolated mature embryos could be stored in WIM for up to 1 week at 4°C without compromising the callus induction efficacy.

### 2.4 *Agrobacterium* inoculation and callus induction

After centrifugation of overnight culture of *Agrobacterium* harbouring desired binary vector in 50 mL Falcon, the supernatant was removed. The pellet was resuspended in 10 mL WIM and an optical density (OD) of 0.4–0.5 at 600 nm was maintained. This *Agrobacterium* solution was aided with 100 µM acetosyringone and placed at 25°C–28°C shaker with 50–80 rpm for 4 h. The WIM was removed from 1.5 mL centrifuge tube carrying wheat mature embryos with the help of pipette and a 1 mL *Agrobacterium* solution was added and placed at room temperature after gently mixing for 5 min. Two batches of transformations were conducted for both pJ32 and pRGEB32 vectors. For each batch, approximately 500 mature embryos were inoculated. The *Agrobacterium* solution was removed with pipette and the mature embryos were dried on sterilized filter paper. The mature embryos were placed on callus induction media (CIM), aided with 100 µM acetosyringone for 3 days in dark at 22°C–24°C, with scutellum side up. The CIM contained 4.4 g L^−1^ MS, 40 g L^−1^ maltose, 10 g L^−1^ glucose, 100 mg L^−1^ myoinositol, 500 mg L^−1^ glutamine, 100 mg L^−1^ casein hydrolysate, 2 g L^−1^ MES, 2 mg L^−1^ Picloram (PhytoTech Labs, KS, United States), 1 mg L^−1^ 2,4-D, and 3.5 g L^−1^ Phytagel (Plant Media, OH, United States) with a pH = 5.8. After 3 days, the mature embryos were transferred to CIM containing 160 mg L^−1^ Timentin (Plant Media, OH, United States) (CIM-T) and placed in dark at 22°C–24°C. After 15 days, the calli were shifted to fresh CIM-T aided with 15 mg L^−1^ Hygromycin (PhytoTech Labs, KS, United States) (CIM-TH). The concentration of hygromycin may vary from cultivar to cultivar. The calli were subculture to fresh CIM-TH after every 14 days.

### 2.5 Shoot regeneration from transgenic calli

After 3 subsequently shifting to CIM-TH, the viable calli were transferred to shoot induction media (SIM) containing 4.4 g L^−1^ MS, 30 g L^−1^ Maltose, 0.5 g L^−1^ MES, 1.25 mg L^−1^ CuSO_4_.5H_2_O, 2 mg L^−1^ Zeatin, 160 mg L^−1^ Timentin, 15 mg L^−1^ Hygromycin, and 4 g L^−1^ Phytagel, with a pH = 5.8. The Petri dishes containing transgenic calli were placed at 24°C ± 1°C with a 16 h photoperiod. The calli were subculture to fresh SIM after every 14 days. In a period of 2–3 weeks, the calli were started turning green and emergence of tiny shoots could be observed. The regeneration and transformation efficiencies were calculated by using methods provided by (Biswal et al., 2023).

### 2.6 Root induction in transgenic plants

After the emergence of shoots, the plantlets with 2–3 cm length were shifted to glass jars containing root induction media (RIM). The RIM contained 4.4 g L^−1^ MS, 30 g L^−1^ Maltose, 160 mg L^−1^ Timentin, 15 mg L^−1^ Hygromycin, and 4 g L^−1^ Phytagel, with a pH = 5.8. The glass jars containing transgenic plantlets were placed at 24°C ± 1°C with a 16 h photoperiod. After a period of 1–2 weeks, emergence of roots could be observed.

### 2.7 Acclimatization and transfer of plantlets to soil

The regenerated plants with proper shoots and roots were removed from glass jars. These plants were shifted to plastic pots, containing a mixture of autoclaved soil and sand, after washing off the media remnants from the roots with tap water. The pots carrying plants were covered with polythene bags for better acclimatization and placed at 24° ± 1°C with a 16 h photoperiod and at 20°C ± 1°C in the dark for 8 h, with 65% humidity and 600 μmol m^−2^ s^−1^ light intensity provided by tungsten bulbs. With an interval of 2 days, the corners of polythene bags were cut followed by complete removal after 6 days.

### 2.8 PCR-based screening of transgenic events and expression analysis of Cas9-positive lines

Plant DNA was extracted by taking fresh leaf samples from putative transgenic plants acquired through tissue culture by using CTAB method. The transgenic events were confirmed via amplification of *hptII* (hygromycin phosphotransferase) gene by using Hygro-F 5′- TCG​TGC​TTT​CAG​CTT​CGA​TG3′ and Hygro-R 5′-GTC​CGT​CAG​GAC​ATT​GTT​GG3′ primers, and Phire Hot Start II DNA Polymerase (Thermo Fisher Scientific, MA, United States), generating a fragment of 520 bp. For the total RNA extraction, fresh leaf sample were collected from positive transgenic plants. Total RNA was extracted from fresh leaves at different growth stages including seedling, tillering, and jointing by using TRIzol Plant RNA isolation kit (Invitrogen, MA, United States). The cDNA was synthesized from total RNA extracted by using the RevertAid First-strand cDNA Synthesis Kit according to the instructions provided by the manufacturer (Thermo Fisher Scientific, MA, United States). To check the relative expression of *TaD27* homeologs and Cas9 transgene under rice (*Oryza sativa*) ubiquitin promoter, quantitative PCR (qPCR) was performed to compare the expression pattern of *TaD27* homeologs and Cas9 gene in GA-wildtype (GA-WT) plants-generated from tissue culture as control, T_0_ mutant (T_0_P3 and T_0_P6), and T_1_ progeny of mutant plants. A qPCR was performed thrice in 15 μL by using the SYBR™ Green PCR Master Mix (Thermo-Fisher Scientific, MA, United States), and primers given in Supplementary Table S1. A ∼200 ng of cDNA was used as the template and the reactions were conducted in triplicates, three biological replicates from each sample and each having three technical replicates. The profile was set as 95°C for 5 min (min); followed by 40 cycles of 95°C for 30 s (sec); 55°C for 30 s; and 72°C for 30 s. Following the completion of the amplification cycles, a melt curve analysis was conducted. The *TaActin* gene (*TraesCS1A02G020500*) was used as an endogenous control for data normalization. The relative fold difference for each sample was calculated through the ΔΔCt method.

### 2.9 DNA sequencing for editing confirmation

For the confirmation of editing in T_0_ plants, the primers were designed 100–200-nt upstream and downstream from the targeted sequence. To check editing in different homeologs of *TaD27* gene, subgenome-specific primers were designed. For the amplification of selected regions of *TaD27-A*, *TaD27-B*, and *TaD27-D* homeologs, TaD27A-F 5′-TAC​AAA​TAT​AAC​CCT​CCC​CC, TaD27B-F 5′-TAA​CAT​CCT​ACA​AAT​ACA​CC3′, and TaD27D-F 5′-GTC​AAA​TCA​TAA​GCT​TAG​GA3′ primers were used, respectively, with a common reverse primer, i.e., TaD27-R 5′-GAA​GCT​TCT​TGA​AGT​TTC. The amplifications were performed by using Platinum™ SuperFi II DNA Polymerase (Thermo Fisher Scientific, MA, United States), and ∼50 ng of genomic DNA as template. After gel documentation, the amplified products were cleaned by using Monarch DNA Cleanup Kit (NEB, MA, United States). The PCR cleanup products were sequenced through Sanger sequencing.

### 2.10 Phenotyping and statistical analysis of phenotypic data

After acclimatization, the regenerated plants were grown in clay pot, bearing 30 cm diameter, outside the growth room in wheat growing season of year 2022–23. The *Tad27* mutant plants (T_0_P3 and T_0_P6) were assessed for phenotypic traits such as number of productive and non-productive tillers per plant, spike length, spikelet per spike, grains per spike, total number of grains per plant, and hundred grains weight in comparison with GA-WT plants. For spike length, spikelet per spike, and grains per spike, data was collected from five different tillers of each T_0_ plant, separately (*n* = 5). Similarly, the T_1_ progenies were also assessed in clay pots in wheat growing season of year 2023–24. The phenotypic data of agronomically significant traits including number of tillers, plant height, spike length, flag leave area, spikelet per spike, grains per spike, total number of grains per plant, and thousand grain weight (extrapolated by measuring weight of hundred seeds) of all the plants including control plants were collected. One-way analysis of variance (ANOVA) was performed for the assessment of extent of variations among the agronomically significant traits of edited and GA-WT plants by using mean values of 15 plants by sowing five seeds each collected from different tillers of T_0_ plants (*n* = 15). The error bars in the bar graphs are ± standard errors (S.E.). Significance differences were assessed with one-way ANOVA *post hoc* Tukey HSD test, where * represents *p* < 0.05 and ** represents *p* < 0.01.

## 3 Results

### 3.1 Designing of guide RNAs in *TaD27* homeologs and amplification of targeted regions

**FIGURE 1:**
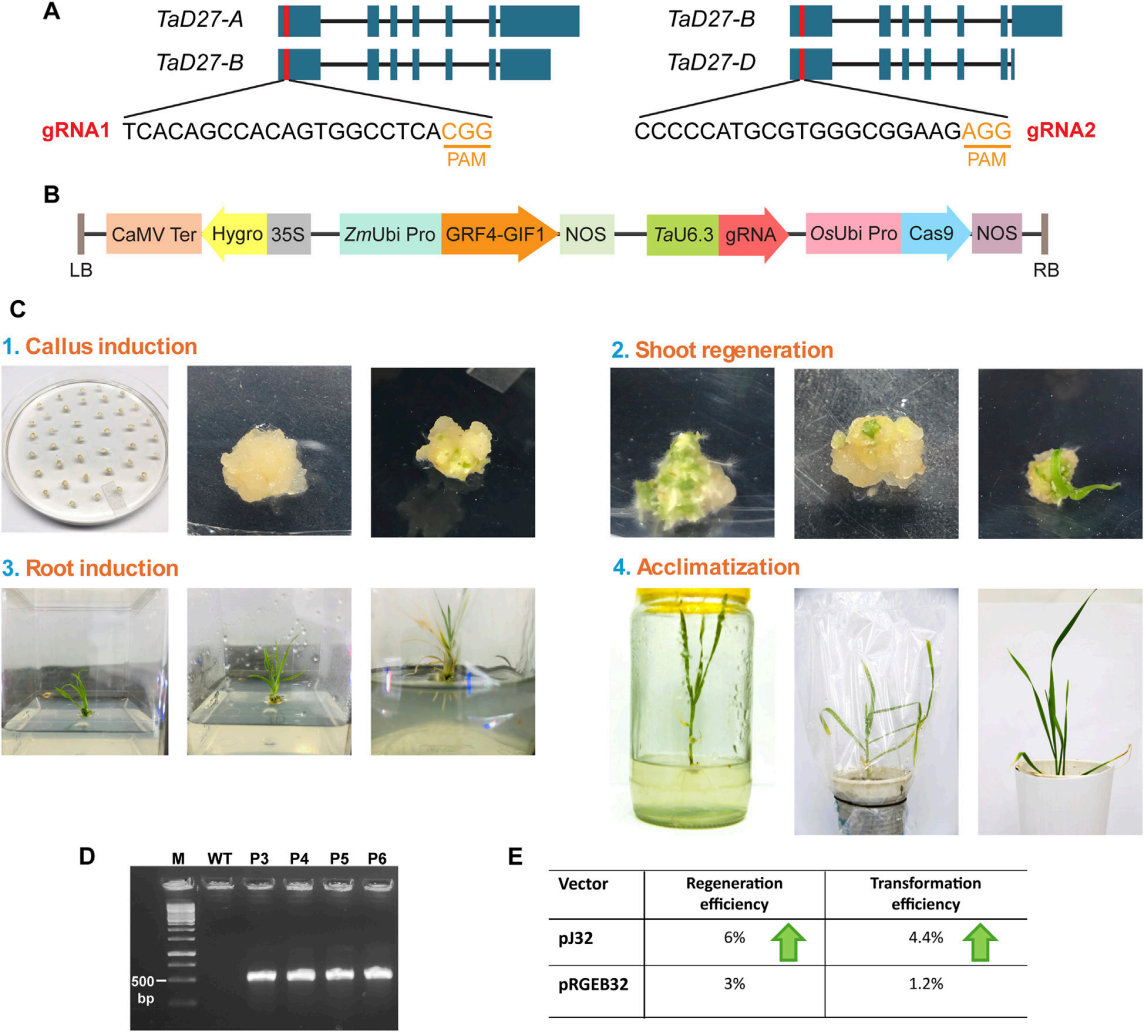
Designing of gRNA and transformation of wheat embryos. **(A)** The position of guide RNAs (gRNAs) designed from the conserved regions of first exons in all the homeologs of *TaD27* gene. The gRNA1 was designed in the conserved regions of *TaD27*-A and *TaD27*-B homeologs and possessed natural polymorphism in *TaD27*-D homeolog in the seed sequence. The gRNA2 was designed in the conserved regions of *TaD27*-B and *TaD27*-D homeologs and possessed natural polymorphism in *TaD27*-A homeolog in the protospacer adjacent motif (PAM) sequence. **(B)** The map of T-DNA cassette in pJ32 vector carrying the *GRF4*-*GIF1* cassette for enhancing regeneration efficiency and *TaU6.3* promoter for the transcription of single gRNA (sgRNA) in the backbone of pRGEB32 vector with *hptII* (hygromycin phosphotransferase) gene for plant selection. **(C)** Wheat tissue culture from mature embryos by using regeneration enhancing cassette (*GRF4*-*GIF1*). (1) Isolation of mature embryos after imbibing the sterilized mature seeds in 5 mgL^−1^ 2,4 D solution for overnight. The embryos were immersed in *Agrobacterium* culture before placing them onto co-cultivation media. Calli generated on callus induction media. (2) Embryogenic calli developed on regeneration media carrying hygromycin selection. (3) Shifting of regenerated shoots in glass jars. Root development of regenerated shoots on root induction media. (4) Transplantation of plantlet with well-developed roots into soil containing pots and covered with plastic bags for better acclimatization. Plastic bags were removed after 3–4 days. **(D)** Confirmation of T-DNA integration in the transgenic events, namely, P3, P4, P5, and P6 plants, via polymerase chain reaction by using primers specific to *hptII* gene, where untransformed GA-WT (WT) plant served as negative control. **(E)** Comparison of regeneration and transformation efficiencies of mature embryos of GA cultivar after *Agrobacterium*-mediated delivery of pJ32 and pRGEB32 vectors.

For the designing of guide RNAs (gRNAs) to target *TaD27* (a negative regulator of tillering), the sequences of three homeologs *TaD27-*A (*TraesCS7A02G418900*), *TaD27-*B (*TraesCS7B02G319100*), and *TaD27-*D (*TraesCS7D02G411500*) were retrieved from the database of the International Wheat Genome Sequencing Consortium, EnsemblPlants (http://plants.ensembl.org/). The genomic sequences of all three homeologs of *TaD27* gene consisted of seven exons and six introns (Figure 1A). The coding sequences of the homeologs were aligned to design sgRNA from conserved regions of first exon. Two independent sgRNAs were designed to generate knockout lines. The first guide RNA (gRNA1) 5′-TCA​CAG​CCA​CAG​TGG​CCT​CA was designed in a conserved region of *TaD27-*A and *TaD27-*B homeologs 26 bp downstream from first codon ATG. For *TaD27-*D a single mismatch was present at 8th position from the protospacer adjacent motif (PAM) in the seed sequence (in case of *Sp*Cas9, a 9–12 nt long sequence upstream to the PAM; 5′-NGG) that might hinder the CRISPR-Cas9-mediated double stranded break (DSB) (Hsu et al., 2013). The second gRNA2 5′-CCC​CCA​TGC​GTG​GGC​GGA​AG was designed in a conserved region of *TaD27-*B and *TaD27-*D homeologs 57 bp downstream from first codon ATG, for *TaD27-*A homeolog the PAM sequence was missing at this region. The gRNAs were cloned into pJ32 vector under *TaU6.3* promoter, separately (Figure 1B).

### 3.2 Tissue culture of wheat mature embryos and *Agrobacterium*-mediated transformation

The final constructs were separately transformed into *Agrobacterium* strain EHA105. Then, the *Agrobacterium*-mediated transformation of mature embryos of GA were conducted (Figure 1C) (Hayta et al., 2021). The overnight imbibition of surface sterilized mature seeds in 5 mg L^−1^ solution of 2,4-D (w/v) at 28°C shaker enhanced the callus induction efficacy of mature embryos on callus induction media (CIM), previously reported by Saeed et al. (2017). All the regenerated plants were selected on hygromycin selection, and none showed morphological abnormality. In addition, the regenerated plants were confirmed via PCR-based screening to detect the integration of T-DNA cassette by using *hptII* specific-primers to amplify hygromycin gene (Figure 1D). Out of 30 regenerated plants from embryos transformed with pJ32 constructs, 22 plants showed positive-PCR results for transgene integration; 13 transgenic events were obtained from sgRNA1 construct and 9 were obtained from sgRNA2 construct. To assess the regeneration efficacy of *GRF4*-*GIF1* cassette, mature embryos were also transformed with pRGEB32 vector, carrying only Cas9 and selection marker (*hptII*) in the T-DNA cassette without any gRNA cloned. Only six plants showed positive PCR out of 15 regenerated plants from embryos transformed with pRGEB32 vector. The pJ32 vector showed higher regeneration (6%) and transformation (4.4%) efficiencies of mature embryos of GA cultivar after *Agrobacterium*-mediated delivery as compared to pRGEB32 vector with 3% and 1.2% regeneration and transformation efficiencies, respectively (Figure 1E; Supplementary Table S2).

### 3.3 Mutation detection in T_0_ plants via sanger sequencing

**FIGURE 2:**
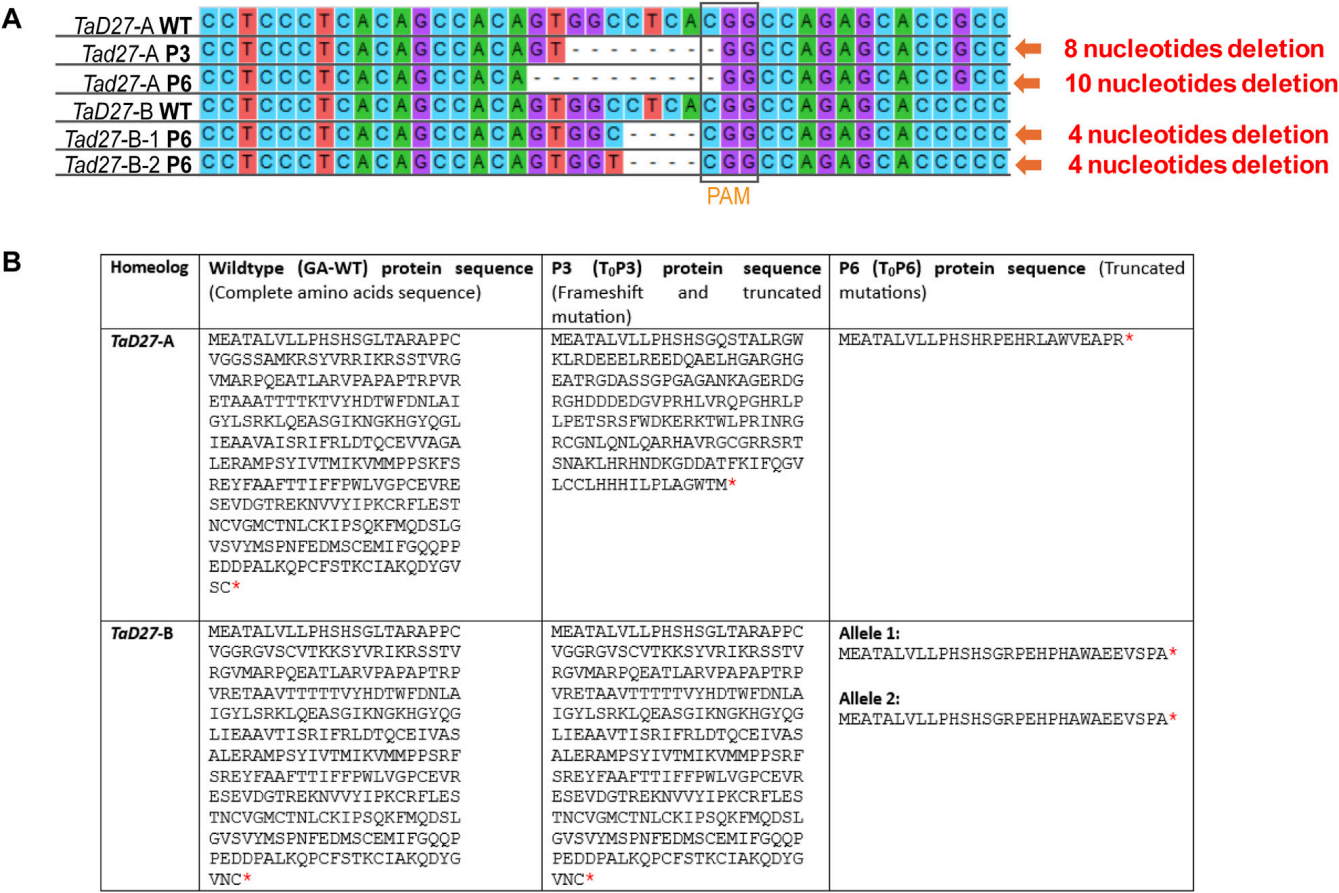
Mutation confirmation in regenerated events through Sanger sequencing. **(A)** Comparison of Sanger sequencing of *TaD27*-A and *TaD27*-B homeologs in GA-wildtype (WT) and T_0_ edited plants, such as T_0_P3 (P3) and T_0_P6 (P6), which shows deleterious at targeted sites, designed according to protospacer adjacent motif (PAM). **(B)** Amino acid sequences of WT and edited *TaD27* homeologs in mutant lines. Red asterisk represents stop codon.

In regenerated T_0_ plants transformed with sgRNA1 construct, mutations in two plants, namely, T_0_P3 and T_0_P6 were identified (Figure 2A). T_0_P3 plant (hereafter referred to as P3) showed mono-allelic/homozygous mutation in a single *TaD27-*A homeolog. While T_0_P6 plant (hereafter referred to as P6) showed mutations in both *TaD27-*A and *TaD27-*B homeologs, with mono-allelic/homozygous mutation in the *TaD27-*A homeolog while bi-allelic/heterozygous mutation in the *TaD27*-B homeolog (Figure 2A; Supplementary Figures S3A, B). Sequencing results showed 8 bp deletion in *TaD27-*A homeolog of P3 (*aaBBDD*) plant while 10 bp and 4 bp deletions in *TaD27-*A and *TaD27-*B homeologs of P6 (*aabbDD*) plant, respectively, where the *TaD27*-B homeolog also exhibited bi-allelic mutation, specifically a C to T substitution (Figure 2A; Supplementary Figures S3A, B). In regenerated plants transformed with sgRNA2 construct, we found no mutation in either *TaD27-*B or *TaD27-*D homeologs in any plant. No evidence of mutagenesis was found in non-targeted *TaD27-*D homeolog in any transgenic plant might be owing to the presence of natural polymorphism at 8th position in the seed sequence of the gRNA (Supplementary Figure S3C). The resulted deletions displayed frameshift and loss-of-function mutations in the targeted homeologs. Owing to frameshift mutations in the first exon, insertion of premature stop codons occurred that resulted in the truncation of proteins encoded by *TaD27*-A homeologs in both P3 and P6 edited plants. Whereas, in case of *TaD27*-B homeolog, the resulted frameshift mutation in P6 edited plant generated bi-allelic edits and both alleles encoded truncated proteins (Figure 2B).

### 3.4 Expression analysis of *TaD27* homeologs and Cas9 transgene

**FIGURE 3:**
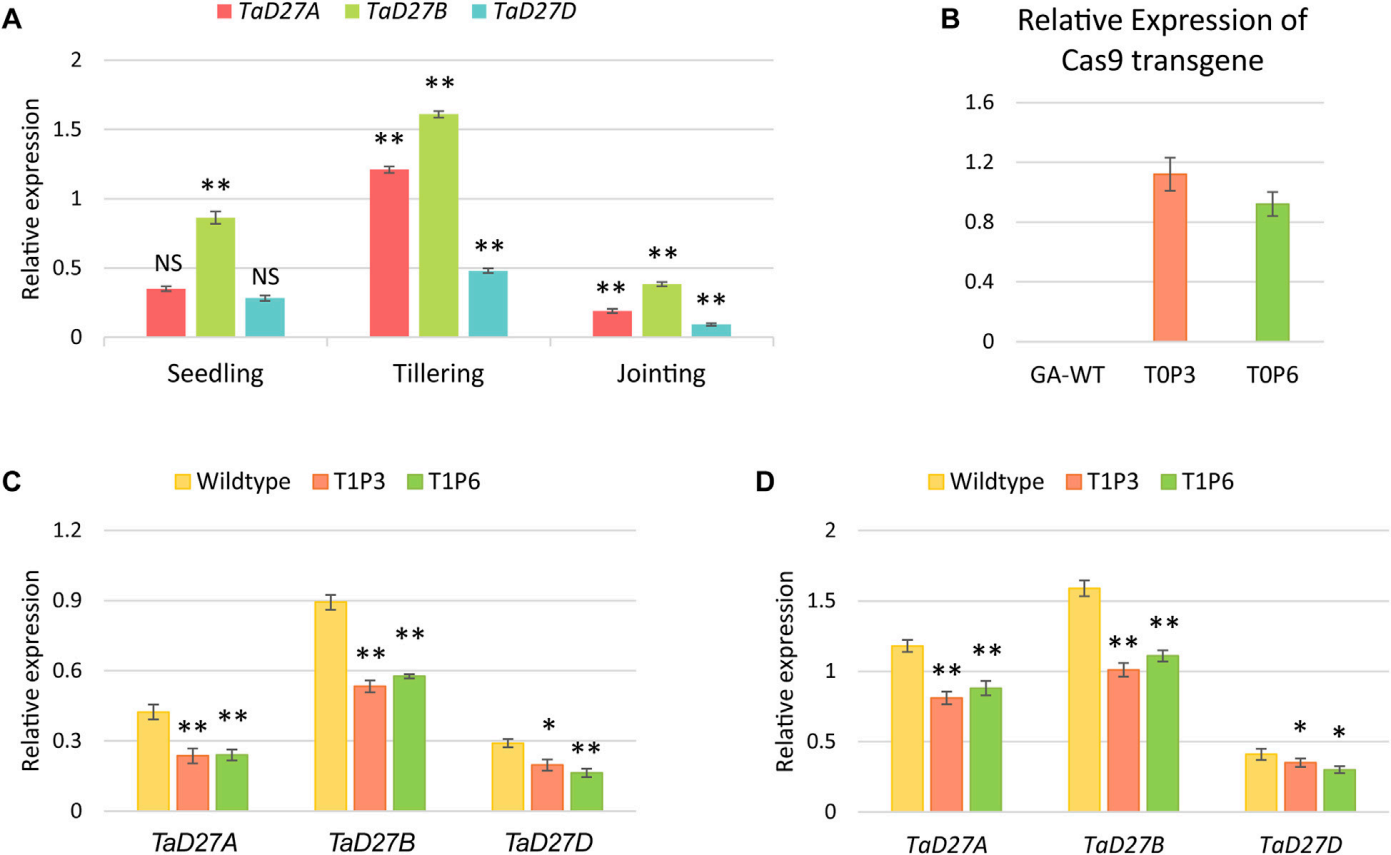
Expression analysis of *TaD27* homeologs and Cas9 transgene. **(A)** Expression patterns of *TaD27* homeologs in GA-2002 cultivar at different growth stages such as seedling, tillering, and jointing. **(B)** Relative expression of Cas9 transgene in T_0_ edited plants. **(C–D)** Relative expression of *TaD27* homeologs in T_1_ generation at seedling and tillering stages, respectively. Data is represented by using mean values (*n* = 3). The error bars in the bar graphs are ± standard errors (S.E.). Significance differences were determined with one-way ANOVA *post hoc* Tukey HSD test; *p < 0.05, **p < 0.01. NS represents no significant difference.

To assess the transcriptional dynamics of *TaD27* gene in GA cultivar, the expression patterns of homeologs of *TaD27* gene were analysed which demonstrated varying notion at different growth stages including seedling, tillering, and jointing (Figure 3A; Supplementary Table S3). All the homeologs showed higher expression at tillering stage, contrary to jointing stage where the lower expression level was observed. Notably, at all stages, the *TaD27*-B homeolog portrayed dominant expression patterns which predicts the role of regulatory signatures such as microRNAs and transcription factors influencing the expression patterns (Supplementary Figures S4, S5). The expression level of Cas9 transgene under the *OsUbi* promoter in the transgenic plants was assessed. The Cas9 expression profile in both T_0_ plants (P3 and P6) showed higher expression level (Figure 3B; Supplementary Table S4). The expression profiles of *TaD27* homeologs were also observed in GA-WT and T_1_ progeny of mutant plants at seedling and tillering stages (Figures 3C, D; Supplementary Tables S5, S6). The expression of targeted homeologs was significantly lower in T_1_ mutants as compared to GA-WT control. These results are consistent with the previous findings involving the transcriptional suppression of a gene due to targeted binding of CRISPR-Cas9-gRNA complex to the endogenous gene (Liu et al., 2016). Akin to the expression profiles in GA-WT plants (Figure 3A; Supplementary Table S3), the expression of *TaD27*-B homeologs was higher compared to *TaD27*-A and *TaD27*-D homeologs in T_1_ mutant lines (Figure 3C; Supplementary Table S5).

### 3.5 Increase in the number of tillers and number of grains in *Tad27* mutant lines

**FIGURE 4:**
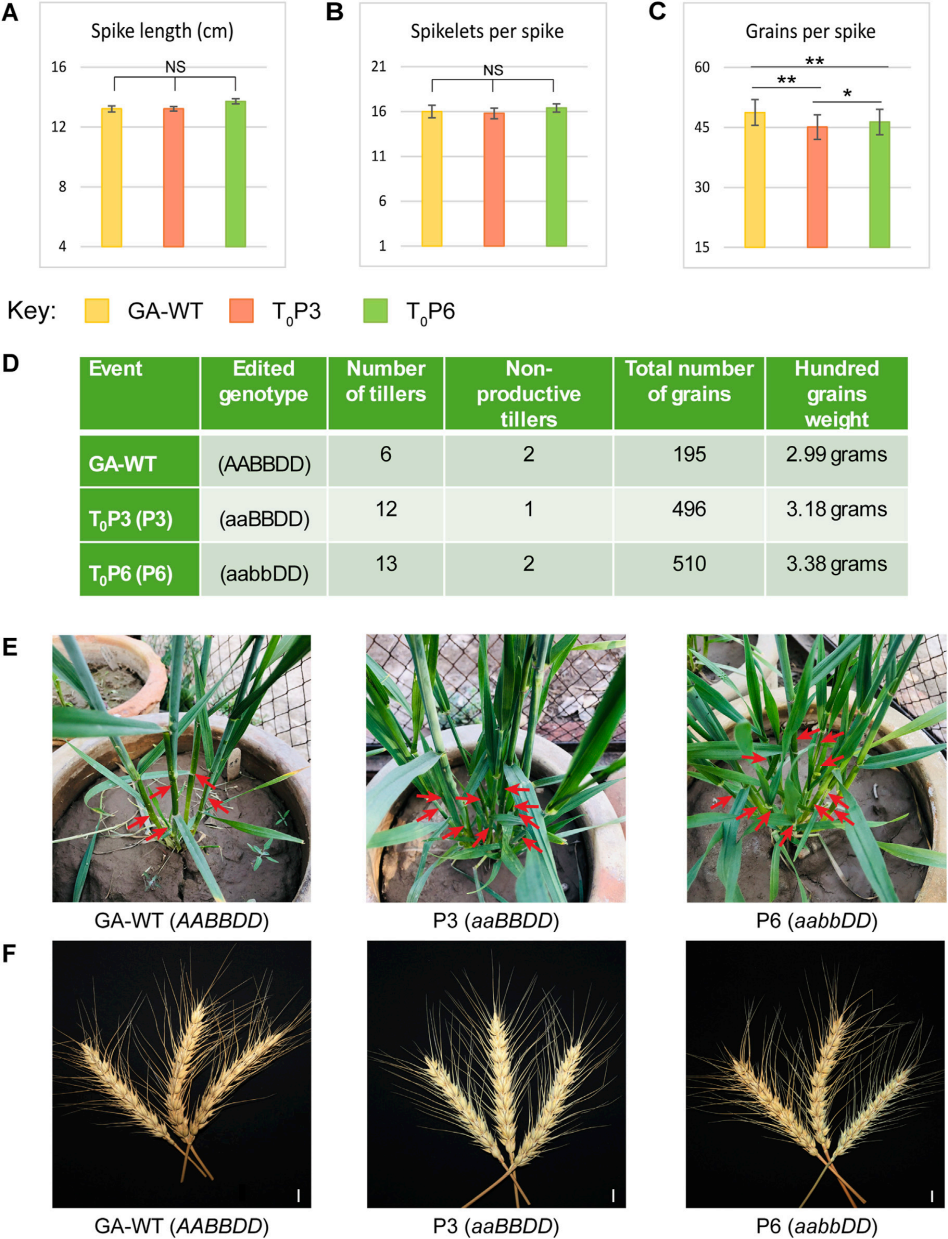
Phenotypic assessment of T_0_ mutant lines. **(A–C)** Phenotypic assessment of the extent of variations among the agronomically significant traits of GA-WT and mutant plants by using mean values (*n* = 5 tillers per plant). The error bars in the bar graphs are ± standard errors (S.E.). Significance differences were determined with one-way ANOVA *post hoc* Tukey HSD test; *p < 0.05, **p < 0.01. NS represents no significant difference. **(D)** Phenotypic assessment of variations among the agronomical traits. **(E)** Morphological comparison of the number of tillers of GA-WT with P3 and P6 mutant plants. **(F)** Morphological comparison of spikes of GA-WT with P3 and P6 mutant plants. Scale bar, 2 cm.

**FIGURE 5:**
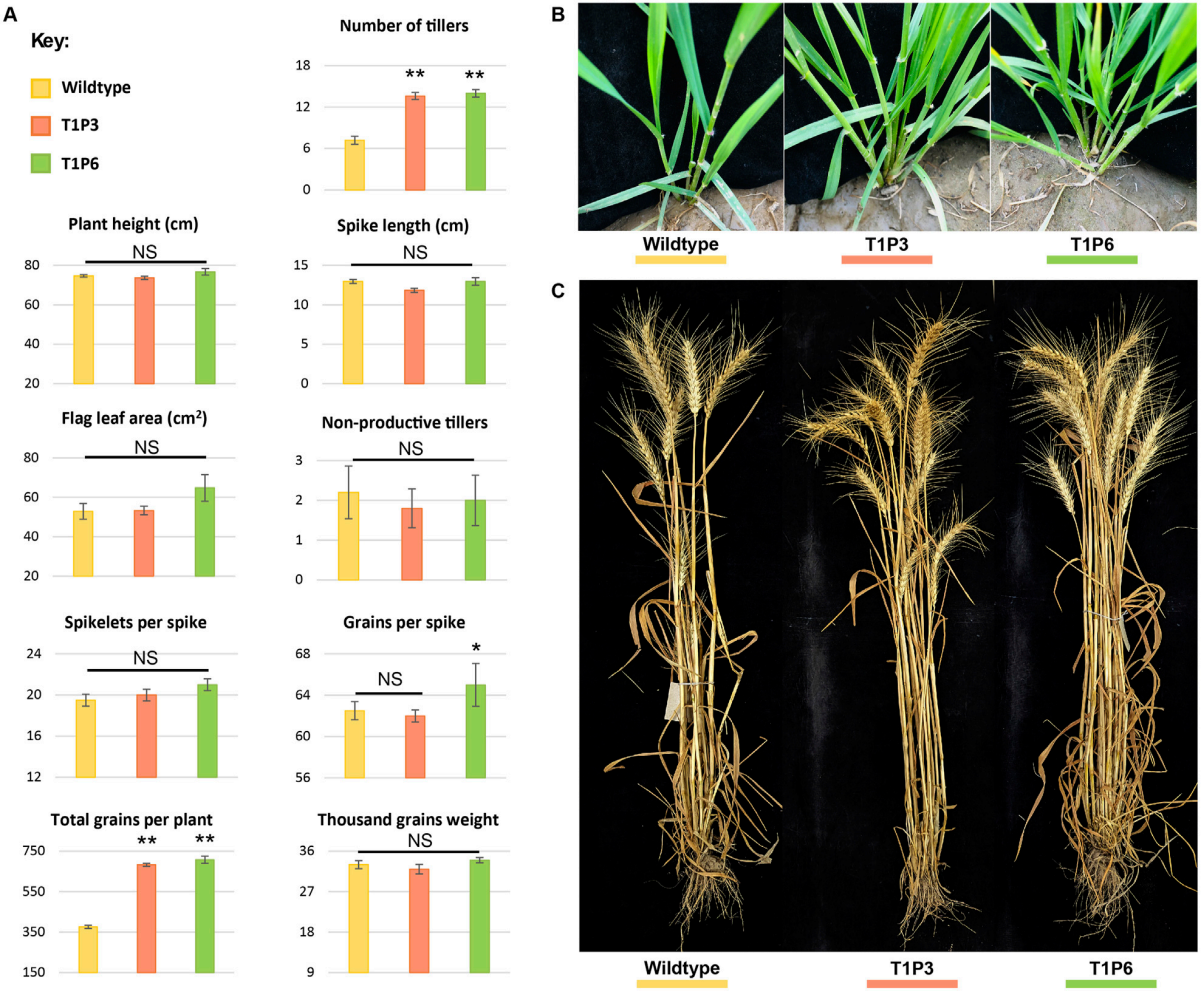
Morphological assessment of wildtype and T_1_ mutant lines. **(A)** Phenotypic assessment of extent of variations among the agronomically significant traits of GA-WT and T_1_ progeny of mutant plants by using mean values (*n* = 15 plants). The error bars in the bar graphs are ± standard errors (S.E.). Significance differences were determined with one-way ANOVA *post hoc* Tukey HSD test; *p < 0.05, **p < 0.01. NS represents no significant difference. **(B)** Morphological assessment of the number of tillers at the tillering stage in GA-WT and T_1_ generation of P3 and P6 plants. **(C)** Morphological assessment of number of tillers at the harvesting stage in GA-WT and T_1_ generation of P3 and P6 plants.

The phenotypic potential of T_0_ and T_1_ generations of *Tad27* mutants (P3 and P6) was evaluated in comparison with GA-WT plants generated from tissue culture as negative control and statistically analysed by employing one-way ANOVA with Tukey’s *post hoc* test to infer the trends (Figures 4, 5). Among the T_0_ mutants, P3 (*aaBBDD*) bearing mutation only in *TaD27*-A homeolog showed no significant change in the spike length and spikelets per spike as compared to GA-WT (Figures 4A, B; Supplementary Table S7), however, a significant (*p* < 0.01) reduction in the number of grains per spike was observed (Figure 4C; Supplementary Table S7). The T_0_ mutant P6 (*aabbDD*), bearing mutations in *TaD27*-A and *TaD27*-B homeologs also demonstrated no significant change in the spike length and spikelets per spike as compared to GA-WT (Figures 4A, B; Supplementary Table S7), however, like P3 mutant a significant (*p* < 0.01) decline in the number of grains per spike was recorded (Figure 4C; Supplementary Table S7). Results demonstrated a significant increase in the number of tillers in both *Tad27* knockout mutants as compared to GA-WT plants (*p* < 0.01). Compared to GA-WT plants, a twofold increase in the number of tillers in single homeolog mutant- P3 and double homeologs mutant- P6 plants were observed (Figure 4D). However, no significant difference was recorded for non-productive tiller. Owing to increase in the number of productive tillers, the total number of grains per plant was also enhanced in P3 and P6 mutants with slight increase in the hundred grain weight compared to GA-WT plants (Figure 4D). These results consolidate the previous findings that *TaD27* gene negatively regulates the number of tillers in wheat (Zhao et al., 2020), and the *Tad27* knockout mutants effectively increased the number of productive tillers without compromising plant stature and spike architecture (Figures 4E, F).

Both P3 and P6 mutant lines were advanced to T_1_ generation to assess the phenotypic performance of their progeny. Similar to T_0_ mutants, the T_1_ progeny of both P3 and P6 mutants demonstrated significant increase in number of productive tillers and total number of grains per plant as compared to GA-WT plants (*p* < 0.01) (Figure 5A; Supplementary Tables S8, S9). While the other parameters such as plant height, flag leaf area, spike length, and spikelets per spike showed non-significant differences (Figure 5A; Supplementary Tables S8, S9). Owing to increase in the number of tillers, a significant increase of 1.8-fold in the number of grains per plant was also observed in *Tad27* mutant lines (*p* < 0.01) (Figure 5A), whereas only P6 mutant line showed higher number of grains per spike compared to P3 and GA-WT plants (*p* < 0.05) (Figure 5A).

In addition, the transgenic events with knockout mutations in single or double homeologs coupled with Cas9-sgRNA-mediated targeted transcriptional suppression offer similar increase in the number of tillers per plant. All the transgenic edited plants showed similar morphology to the GA-WT plants without any indication of dwarfing or phenotypic deformity (Figures 5B, C). Consistent with previous findings, *TaD27* might not regulate plant height in wheat (Zhao et al., 2020). Taken together, these results show that knockout mutation in *TaD27* gene enhances number of productive tillers, subsequently increasing grain yield per plant.

## 4 Discussion

The CRISPR-Cas9-mediated targeted mutagenesis has been employed in various food security crops including rice, maize, and wheat, to generate knockout mutants to improve agronomically significant traits such as number of grains per plant (Zhu et al., 2020). In wheat, different yield-related negative regulators have been targeted to increase number of grains and grain weight in different cultivars (Shan et al., 2014; Zhang et al., 2019), but there is no report of development of high tillering mutant lines through CRISPR-Cas9-mediated mutagenesis in *TaD27* gene. In this study, the development of high tillering wheat lines is reported by targeting a tillering negative regulator, known as *DWARF 27*, that controls auxiliary bud branching through biosynthesis of SLs. The D27 regulates the MORE AXILLARY GROWTH1 (MAX1) pathway that involves conversion of carlactone (CL) to carlactonic acid (CLA), which is then converted into a methylated compound known as methyl carlactonate (MeCLA) (Waters et al., 2017). In *Arabidopsis*, MeCLA and its derived product, generated by the activity of LATERAL BRANCHING OXIDOREDUCTASE (LBO), demonstrates suppression of shoot branching (Supplementary Figure S1) (Brewer et al., 2016).

The results in this study showed that CRISPR-Cas9-mediated targeted mutagenesis in the mature embryos of elite cultivar of wheat offers rapid crop improvement as compared to conventional breeding which conflates distinct genotypes causing a linkage drag of unwanted traits. The gRNA1 construct targeting *TaD27-*A and *TaD27-*B homeologs, simultaneously, generated similar phenotypic results in P3 (*aaBBDD*) and P6 (*aabbDD*) mutant lines by introducing frameshift mutation which resulted in truncation of encoded proteins. The resulted phenotypes of edited events showed no discrepancies or any additive effect owing to single or double mutations in different homeologs, i.e., *TaD27-*A and *TaD27-*B. Irrespective to homeolog-specific single or double mutations, no significant differences were recorded for other parameters such as plant height, flag leaf area, spike length, and spikelet per spike. Of note, enhanced number of productive tillers increased number of grains per plant, eventually increasing wheat yield. Consistent with the previous findings, this similar phenotypic behaviour between the P3 (*aaBBDD*) and P6 (*aabbDD*) mutant lines might be a result of suppressed transcriptional expression of both *TaD27-*A and *TaD27-*B homeologs due to the binding of CRISPR-Cas9-sgRNA complex to the targeted DNA sites and sterically inducing transcriptional repression (Liu et al., 2016; Didovyk et al., 2016; Gilbert et al., 2013; Piatek et al., 2015). Similar to a previously reported study on RNAi-mediated knockdown of *TaD27*-B, our results also emphasize the concept that disruption of D27 does not induce dwarfing in wheat but affects the biosynthesis of SLs, which are involved in inhibiting the outgrowth of axillary buds or shoot branching (tillers) (Zhao et al., 2020). Conversely, SLs induce dwarfing in rice and *Arabidopsis* by regulating the *Teosinte branched1* (*TB1*) gene (Aguilar-Martínez et al., 2007; Lin et al., 2009), whereas Zhao et al. (2020) reported no significant changes either in the expression of *TaTB1* genes nor in the plant height when supplemented with exogenous application of SLs (GR24) in *TaD27*-B-RNAi plants (Zhao et al., 2020). Being a crucial phytohormone, the SLs signalling pathways also play substantial role in regulating plant growth and development through regulatory elements such as transcription factors (Guo et al., 2013). The loss-of-function mutation in *TaD27* gene disrupts the SLs biosynthesis and SLs-mediated signalling pathways, subsequently, this decline reduces the impact of SLs-mediated inhibition of branching. Moreover, the reduction in SLs biosynthesis also demonstrated an increase in the auxin synthesis and transcription level of auxin-signalling related genes (Zhao et al., 2020). Therefore, a trade-off can be established between two phytohormones, resulting in the reduction of SLs-the inhibitors of axillary bud outgrowth and a boost in auxins-the inducers of axillary bud outgrowth.

Furthermore, the co-transformation of *GRF4*-*GIF1* cassette under single T-DNA cassette also played a substantial role in enhancing the regeneration efficiency in multiple elite cultivars of wheat and can be segregated out as a single T-DNA unit along with CRISPR-Cas9 system (Debernardi et al., 2020; Biswal et al., 2023). The major bottleneck in plant tissue culture is limited regeneration efficiency which has been addressed by overexpression of distinct developmental regulators or morphogenic regulators such as *WUSCHEL*, *BABY BOOM*, *LEAFY COTYLEDON 1*, and *LEAFY COTYLEDON 2* which promote somatic embryogenesis and shoot regeneration in monocots and eudicots (Lotan et al., 1998; Stone et al., 2001; Boutilier et al., 2002; Zuo et al., 2002). The GRF and GIF proteins interact to form a complex and promote various cellular functions such as cell proliferation and tissue development (Debernardi et al., 2014). Therefore, the expression of *GRF4-GIF1* chimera under single promoter (*ZmUBI*) generated a fusion complex which substantially increases regeneration process by influencing cell proliferation and embryogenesis (Debernardi et al., 2020). In this study, the deployment of *GRF4-GIF1* chimera cassette carrying construct (pJ32) showed a 6% regeneration efficiency as compared to 3% regeneration efficiency with a construct without *GRF4-GIF1* chimera cassette (pRGEB32) in elite cultivar of hexaploid wheat. Based on these results, targeted mutagenesis can be acquired by employing CRISPR-Cas9 machinery and higher regeneration frequency with developmental regulators (*GRF4*-*GIF1*) in other elite cultivars or recalcitrant genotypes of wheat. This can also bypass years of backcrossing for the introgression of desired genes through conventional breeding practices. However, advancement of these lines into subsequent generations would allow the acquisition of transgene-free mutant plants to generate seeds which can be commercially available for farmers.

In conclusion, a knockout mutation in *TaD27* gene conferred grain-yield improvement in hexaploid bread wheat by increasing the number of productive tillers per plant. Moreover, these findings can also be translated into other commercially cultivated wheat cultivars, bearing agronomically significant traits such as resistance to crucial pathogens or tolerance against drought or salt stress. This strategy can serve as a potential candidate for genome editing mediated “Second Green Revolution” in wheat. The combination of *TaD27* gene, a negative regulator of tillering, with other yield-related negative regulators, such as *TaGW2*-negative regulator of grain weight (Shan et al., 2014) and *TaCKX2.1*-negative regulator of number of grains (Zhang et al., 2019), can be envisaged as a potential strategy for multiplex genome editing (Li et al., 2021) to improve wheat yield.

## Data Availability

The original contributions presented in the study are included in the article/Supplementary Material, further inquiries can be directed to the corresponding author.
