# Supplementary material for: Knockout mutation in TaD27 enhances number of productive tillers in hexaploid wheat
Source: Front Genome Ed. 2024 Oct 14;6:1455761. doi: 10.3389/fgeed.2024.1455761 (PMC11513295; doi:10.3389/fgeed.2024.1455761)
Supplement: Supplementary file 1 [file DataSheet1.docx]

Supplementary Material

**Knockout mutation in *TaD27* enhances number of productive tillers in hexaploid wheat**

Muhammad Jawad Akbar Awan, Imran Amin, Awais Rasheed, Nasir A. Saeed, and Shahid Mansoor^*^

# Supplementary Figures and Tables

## Supplementary Table

**Supplementary Table 1. List of primers for expression analyses via quantitative PCR.**

| **Sr. #** | **Name** | **EnsemblPlant Accession** | **Sequence 5’-3’** |
| --- | --- | --- | --- |
| 1 | TaActin-qF | *TraesCS1A02G020500* | ATGGCTGACGGTGAGGACAT |
| 2 | TaActin-qR |  | ATGCTAGGGAAAACAGCCCT |
| 3 | D27A-qF | *TraesCS7A02G418900* | ATGAAGAGGAGCTACGTGAG |
| 4 | D27A-qR |  | TGTCGTGGTACACCGTCTTC |
| 5 | D27B-qF | *TraesCS7B02G319100* | GTGGGCGGAAGAGGTGTCTC |
| 6 | D27B-qR |  | TCGTCGTCGTGACCGCGGCA |
| 7 | D27D-qF | *TraesCS7D02G411500* | TCATGGCGAGGCCACAAGAA |
| 8 | D27D-qR |  | GTTTTCCGTTCTTTATCCCG |
| 9 | Cas9-q-F |  | CGAAGAGGGCATCAAAGAGC |
| 10 | Cas9-q-R |  | TCTGAGGCACGATATGGTCC |

**Supplementary Table 2. Comparison of regeneration and transformation efficiencies of pRGEB32 and pJ32 vectors.**

| **Constructs** | **No. of batches** | **Number of embryos *Agro*-inoculated per batch** | **Number of regenerated plants** | **Regeneration efficiency** | **Number of transgenic events** | **Transformation efficiency per batch** |
| --- | --- | --- | --- | --- | --- | --- |
| **pRGEB32** | 2 | 250 | 15 | 3% | 6 | 1.2% |
| **pJ32 (gRNA1 and gRNA2)** | 2 | 250 | 30 | 6% | 22 | 4.4% |

**Supplementary Table 3. Gene expression analysis of *TaD27* homeologs at different growth stages in GA-2002 cultivar.**

| **Stage** | ***TaD27*A** | ***TaD27*B** | ***TaD27*D** |
| --- | --- | --- | --- |
| **Seedling** | 0.35±0.01 | 0.86±0.04 | 0.28±0.02 |
| **Tillering** | 1.21±0.02 | 1.61±0.02 | 0.48±0.01 |
| **Jointing** | 0.19±0.01 | 0.38±0.01 | 0.09±0.01 |

± represents standard error. The relative fold difference for each sample was calculated through the ΔΔCt method.

**Supplementary Table 4. Gene expression analysis of Cas9 in T_0_ mutant plants.**

| **Plants** | **Cas9** |
| --- | --- |
| **GA-WT** | 0.00 |
| **T_0_P3** | 1.12±0.11 |
| **T_0_P6** | 0.92±0.08 |

± represents standard error. The relative fold difference for each sample was calculated through the ΔΔCt method.

**Supplementary Table 5. Gene expression analysis of *TaD27* homeologs at seedling stage in GA-WT and T_1_ mutant progeny.**

| **Plants** | ***TaD27*A** | ***TaD27*B** | ***TaD27*D** |
| --- | --- | --- | --- |
| **GA-WT** | 0.42±0.03 | 0.89±0.03 | 0.29±0.02 |
| **T_1_P3** | 0.23±0.03 | 0.53±0.02 | 0.19±0.01 |
| **T_1_P6** | 0.24±0.01 | 0.57±0.02 | 0.16±0.01 |

± represents standard error. The relative fold difference for each sample was calculated through the ΔΔCt method.

**Supplementary Table 6. Gene expression analysis of *TaD27* homeologs at tillering stage in GA-WT and T_1_ mutant progeny.**

| **Plants** | ***TaD27*A** | ***TaD27*B** | ***TaD27*D** |
| --- | --- | --- | --- |
| **GA-WT** | 1.18±0.04 | 1.59±0.04 | 0.41±0.05 |
| **T_1_P3** | 0.81±0.05 | 1.01±0.04 | 0.35±0.04 |
| **T_1_P6** | 0.88±0.04 | 1.11±0.03 | 0.30±0.02 |

± represents standard error. The relative fold difference for each sample was calculated through the ΔΔCt method.

**Supplementary Table 7.** **Phenotypic data of T_0_ mutant plants.**

| **Plants** | **Spike Length (Inch)** | **Spikelet per spike** | **Grains per spike** |
| --- | --- | --- | --- |
| **GA-WT** | 13.21±0.20 | 16.00±0.71 | 48.75±3.20 |
| **T_0_P3** | 13.20±0.15 | 15.80±0.58 | 45.09±3.09 |
| **T_0_P6** | 13.71±0.17 | 16.40±0.45 | 46.36±3.18 |

± represents standard error.

**Supplementary Table 8. Phenotypic data of T_1_ generation.**

| **Plants** | **Number of tillers** | **Plant height (cm)** | **Spike length (cm)** | **Flag leaf area (cm^2^)** | **Non-productive tillers** |
| --- | --- | --- | --- | --- | --- |
| **GA-WT** | 7.2±0.58 | 74.67±0.62 | 12.95±0.25 | 52.90±3.97 | 2.2±0.66 |
| **T_1_P3** | 13.6±0.50 | 73.66±0.80 | 11.83±0.26 | 53.29±2.19 | 1.8±0.48 |
| **T_1_P6** | 14±0.54 | 76.70±1.68 | 12.95±0.47 | 64.77±6.72 | 2±0.63 |

± represents standard error.

**Supplementary Table 9. Phenotypic data of T_1_ generation.**

| **Plants** | **Number of spikelets per spike** | **Number of grains per spike** | **Thousand grains weight (g)** | **Total grains per plant** |
| --- | --- | --- | --- | --- |
| **GA-WT** | 19.5±0.56 | 62.5±0.88 | 33±0.90 | 375.66±7.88 |
| **T_1_P3** | 20±0.55 | 62±0.57 | 32±1.04 | 682.66±7.50 |
| **T_1_P6** | 21±0.57 | 65±2.08 | 34±0.57 | 707.33±17.89 |

± represents standard error.

**Supplementary Table 10. Wheat inoculation media (WIM) recipe.**

| **Ingredients** | **Concentration** |
| --- | --- |
| **Murashige and Skoog (MS)- Salts** | 0.44 gL^-1^ |
| **Glucose** | 10 gL^-1^ |
| **MES** | 2 gL^-1^ |
| **Silwet L-77** | 0.05 % |
| **Acetosyringone** | 100 μM |

**Supplementary Table 11. Callus induction media (CIM) recipe.**

| **Ingredients** | **Concentration** |
| --- | --- |
| **Murashige and Skoog (MS)- Salts** | 4.4 gL^-1^ |
| **Maltose** | 40 gL^-1^ |
| **Glucose** | 10 gL^-1^ |
| **Myoinositol** | 100 mgL^-1^ |
| **Glutamine** | 500 mgL^-1^ |
| **Casein hydrolysate** | 100 mgL^-1^ |
| **MES** | 2 gL^-1^ |
| **Picloram** | 2 mgL^-1^ |
| **2,4 D** | 1 mgL^-1^ |
| **Acetosyringone** | 100 μM |
| **Phytagel** | 3.5 gL^-1^ |
| **pH** | 5.8 |

**Supplementary Table 12. Callus induction media aided with timentin (CIM-T) recipe.**

| **Ingredients** | **Concentration** |
| --- | --- |
| **Murashige and Skoog (MS)- Salts** | 4.4 gL^-1^ |
| **Maltose** | 40 gL^-1^ |
| **Glucose** | 10 gL^-1^ |
| **Myoinositol** | 100 mgL^-1^ |
| **Glutamine** | 500 mgL^-1^ |
| **Casein hydrolysate** | 100 mgL^-1^ |
| **MES** | 2 gL^-1^ |
| **Picloram** | 2 mgL^-1^ |
| **2,4 D** | 1 mgL^-1^ |
| **Timentin** | 160 mgL^-1^ |
| **Phytagel** | 3.5 gL^-1^ |
| **pH** | 5.8 |

**Supplementary Table 13.** **Shoot induction media (SIM) recipe.**

| **Ingredients** | **Concentration** |
| --- | --- |
| **Murashige and Skoog (MS)- Salts** | 4.4 gL^-1^ |
| **Maltose** | 30 gL^-1^ |
| **MES** | 0.5 gL^-1^ |
| **CuSO_4_.5H_2_O** | 1.25 mgL^-1^ |
| **Zeatin** | 2 mgL^-1^ |
| **Timentin** | 160 mgL^-1^ |
| **Hygromycin** | 15 mgL^-1^ |
| **Phytagel** | 4 gL^-1^ |
| **pH** | 5.8 |

**Supplementary Table 14.** **Root induction media (SIM) recipe.**

| **Ingredients** | **Concentration** |
| --- | --- |
| **Murashige and Skoog (MS)- Salts** | 4.4 gL^-1^ |
| **Maltose** | 30 gL^-1^ |
| **Timentin** | 160 mgL^-1^ |
| **Hygromycin** | 15 mgL^-1^ |
| **Phytagel** | 4 gL^-1^ |
| **pH** | 5.8 |

## Supplementary Figures


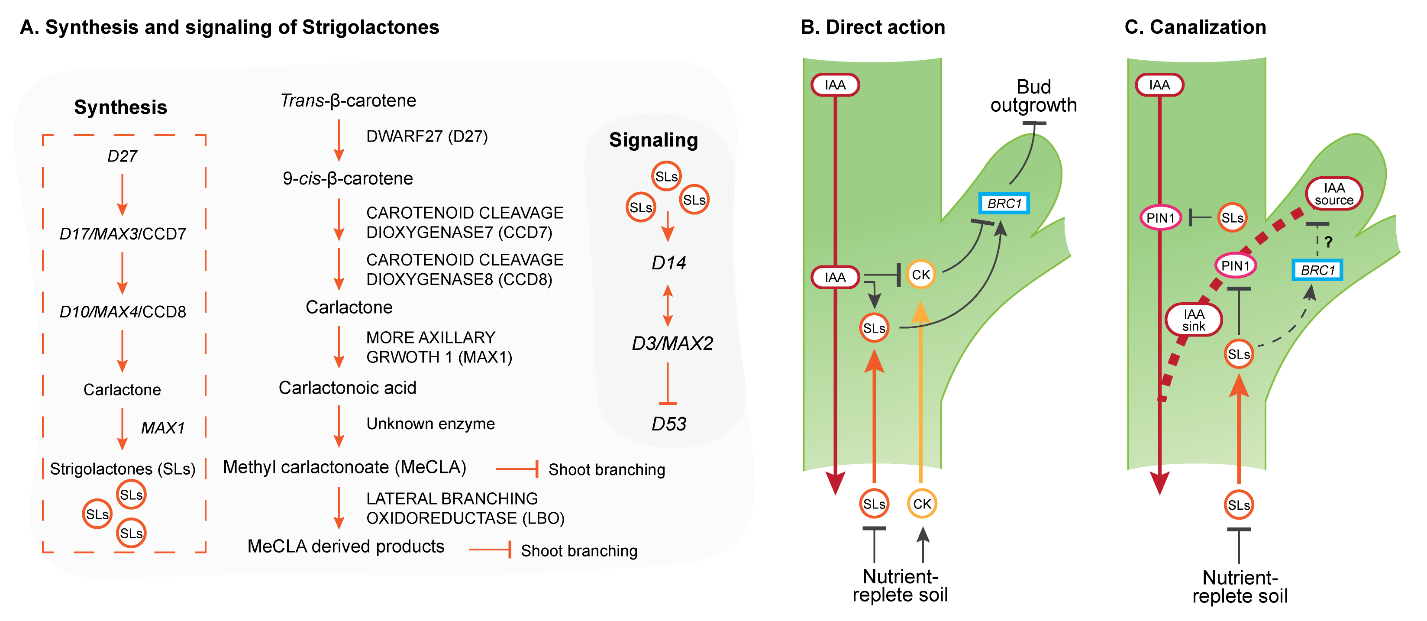


**Supplementary Figure 1. Strigolactones (SLs) biosynthesis and signaling pathways.**

(A) Initiation of biosynthesis pathway of SLs by the activity of DWARF27 (D27) enzyme, followed by the activity of various enzymes. SLs play a crucial role in activating D14 to recruit D3/MAX2 and D53. The D53 is polyubiquitinated and degraded by the activity of proteosome. (B) In the direct-action model, the root-derived SLs promote the expression of transcription factors such as BRC1 that negatively regulates bud outgrowth. In contrast, root-derived CKs inhibit the expression of BRC1 and eventually promote bud outgrowth. The hormonal activity of SLs and CKs in the bud might be modulated by the local level of auxin (IAA). (C) In the canalization model, the bud outgrowth is modulated by capacity of the bud to export auxin. Bud accommodating high concentration of auxin (the source) exports it to the low concentration carrying stem (the sink) with the help of PIN1 transporter. SLs modulate the export of auxins from source to sink by inhibiting the activity of PIN1 in bud as well as in stem. Moreover, SLs also influence the bud outgrowth via BRC1 expression in some species (represented by dashed lines). The availability of soil nutrients impacts the production of root-derived hormones in both models. Abbreviations: IAA, indole-3-acetic acid; BRC1, BRANCHED1; SLs, strigolactones; CK, cytokinin; PIN1, PIN-FORMED1. (B and C) Adopted from: Waters, M. T., Gutjahr, C., Bennett, T., & Nelson, D. C. (2017). Strigolactone signaling and evolution. Annual review of plant biology, 68, 291-322.

**
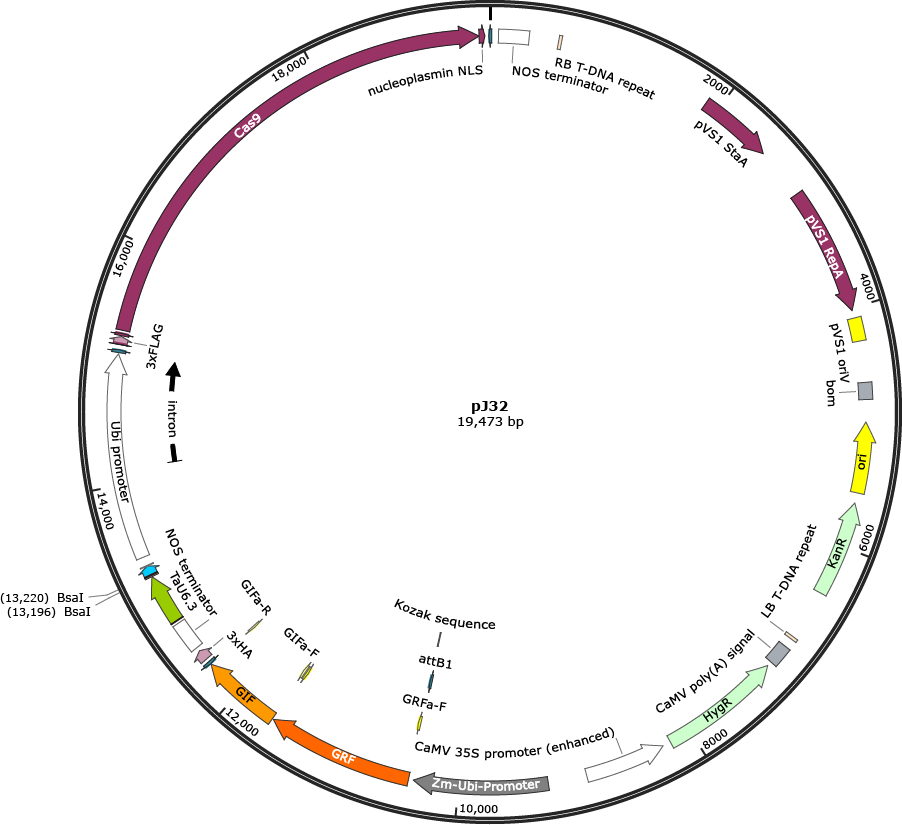
**

**Supplementary Figure 2.** pJ32 vector map- A binary vector with a size of 19,473 nucleotides. pJ32 contains kanamycin resistance for cloning of gRNA in bacteria and hygromycin resistance for the selection of transgenic events.


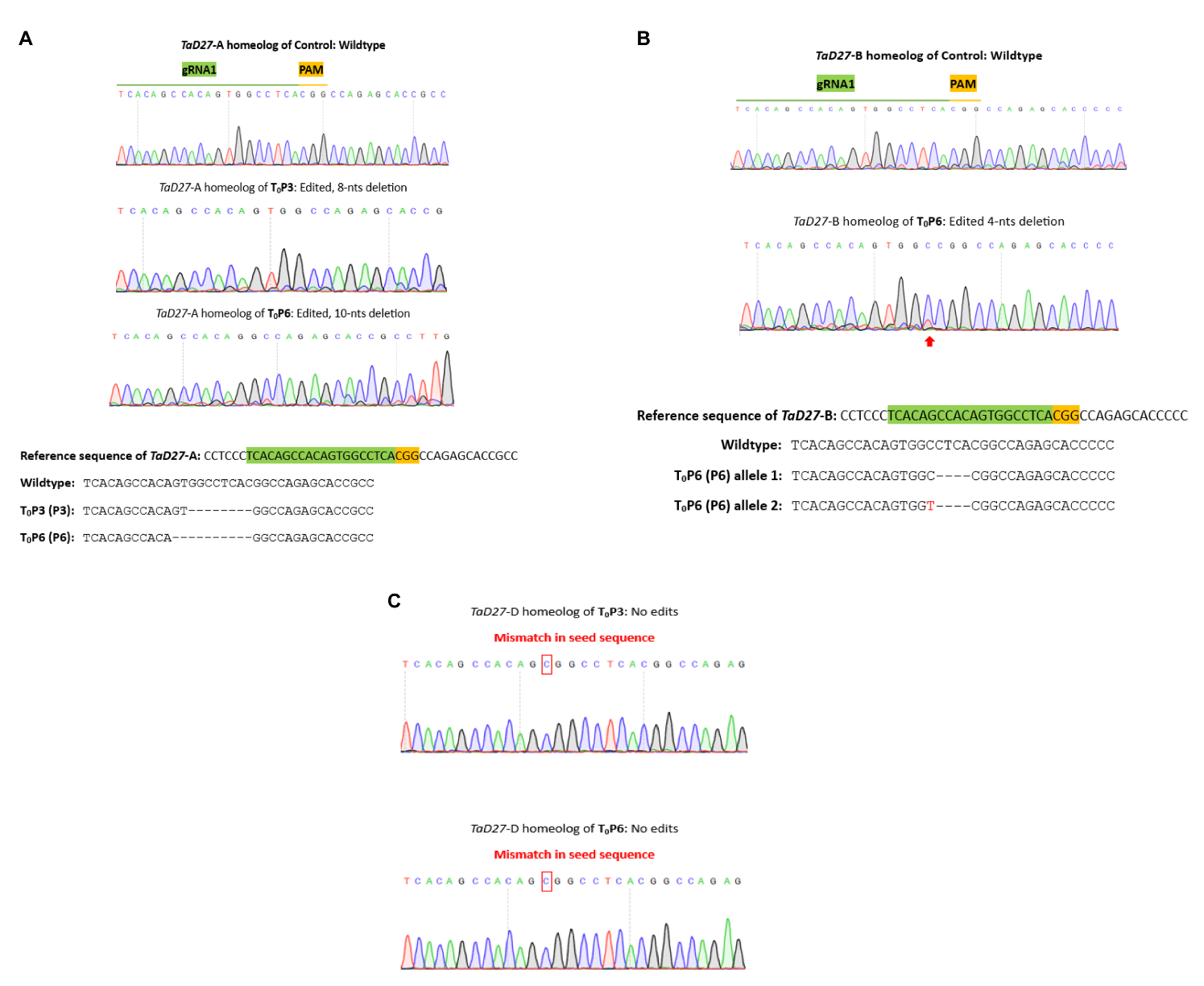


**Supplementary Figure 3.** Sanger sequencing of results of edited plants. (A) Comparison of Sanger sequencing chromatograms of *TaD27*-A homeolog in WT with P3 and P6 edited plants. Green line represents the position of guild RNA (gRNA), and orange line represents the position of PAM. Analysis of targeted mutations in *TaD27*-A homeolog in WT and edited plants (P3 and P6). 8 bp deletion in P3 edited plant and 10 bp deletion in P6 plant were observed. All the mutations observed were homozygous. (B) Comparison of Sanger sequencing chromatograms of *TaD27*-B homeolog in WT and P6 edited plant. Red arrow represents heterozygous mutation. Analysis of targeted mutations in *TaD27*-B homeolog in WT and P6 edited plant. 4 bp deletion in both alleles of *TaD27*-B were observed in P6 plant. However, mutation observed was heterozygous (highlighted in red). (C) Sanger sequencing chromatograms of *TaD27*-D homeolog show no alterations in P3 and P6 edited plants. Red box represents the presence of mismatch at 8th position upstream from the PAM in seed sequence of gRNA.


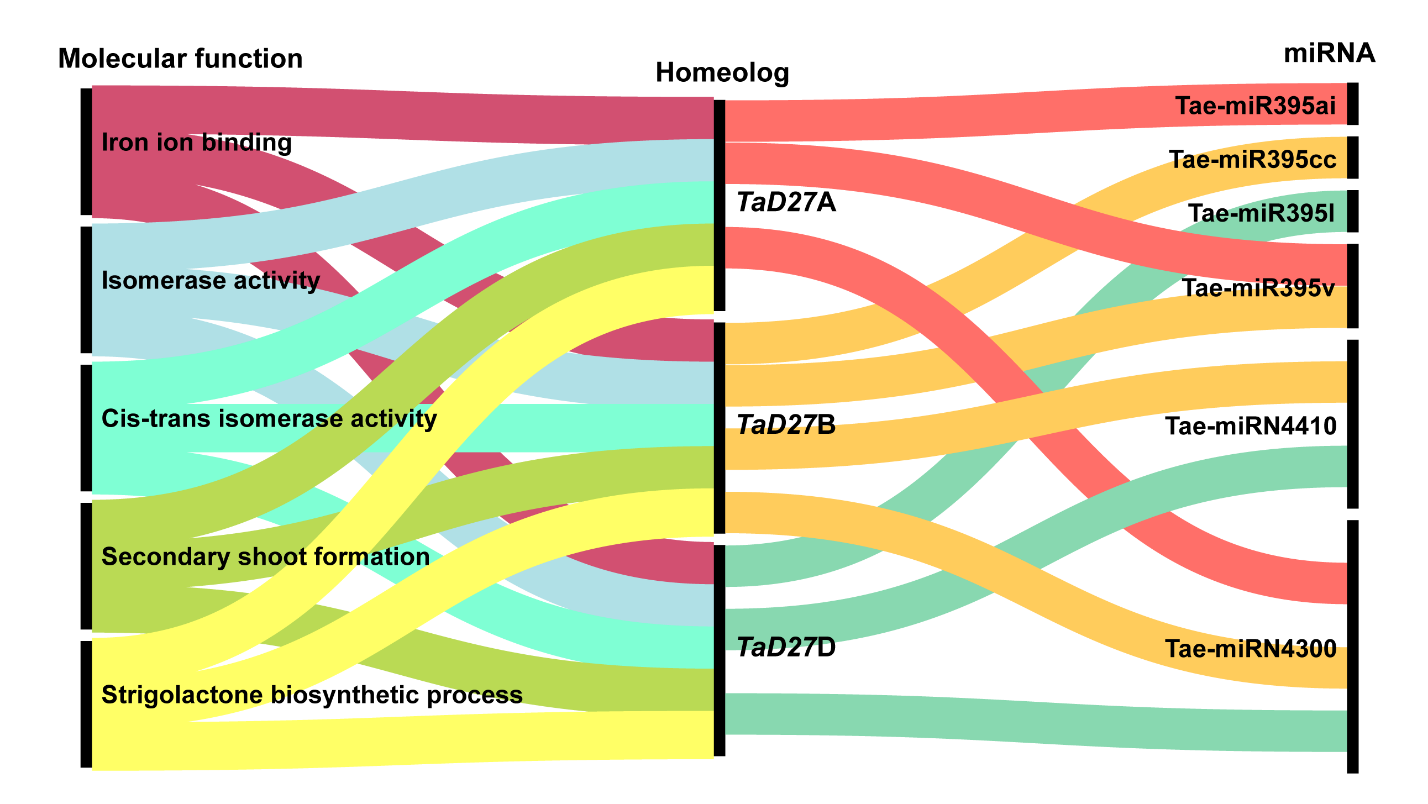


**Supplementary Figure 4.** Illustration of gene ontology (GO) of *TaD27* homeologs and microRNA (miRNA) targeting *TaD27* homeologs. GO for functional annotation was analyzed by using online source DAVID website (<https://david.ncifcrf.gov>). Genomic sequences of *TaD27* homeologs were retrieved via the psRNATarget database (<http://plantgrn.noble.org/psRNATarget/>) to characterize potential miRNAs. Graphical illustration was created by using online source RAWGraphs (<https://www.rawgraphs.io/>).


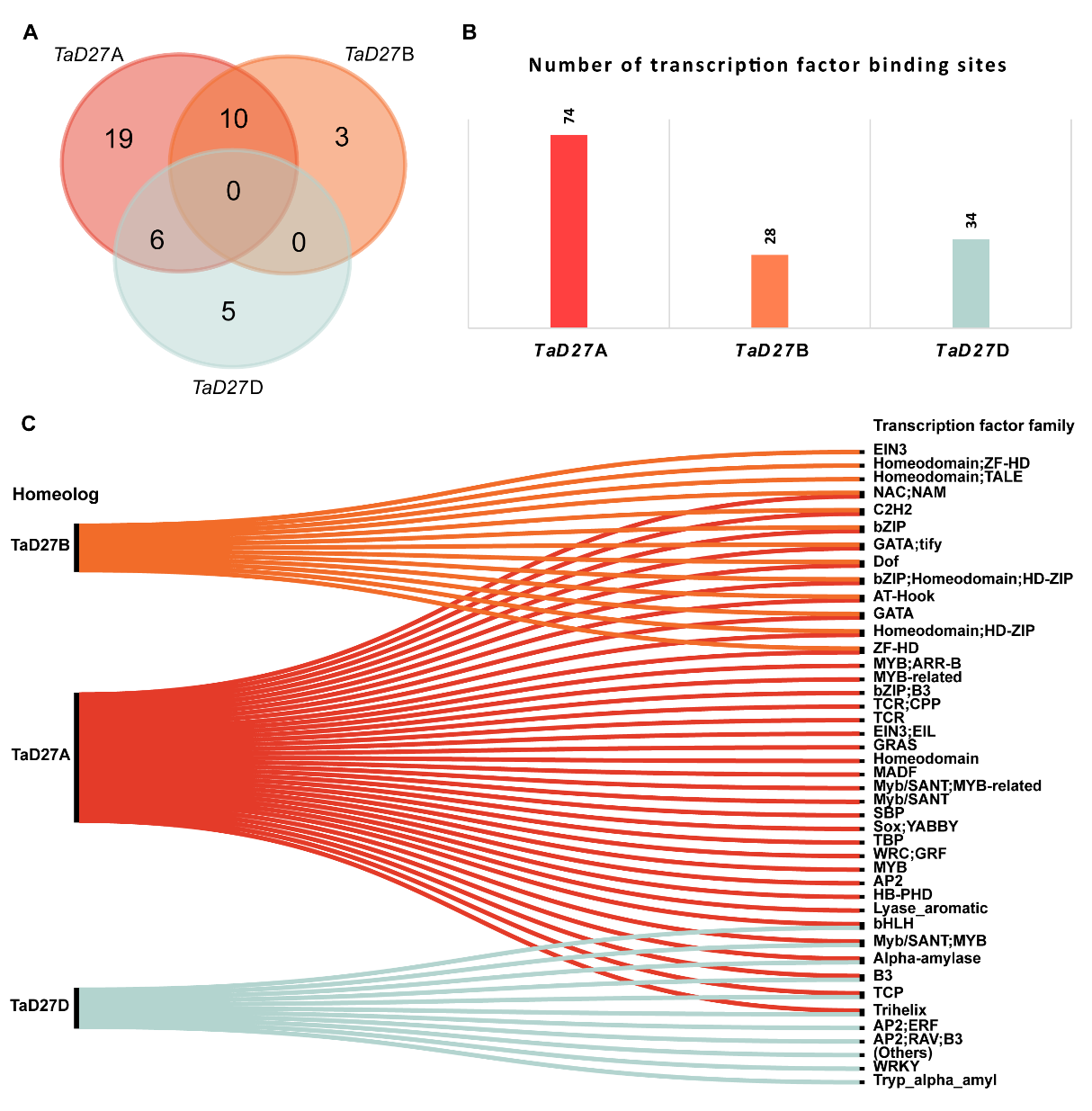


**Supplementary Figure 5. Transcription factor binding sites analysis of *TaD27* homeologs.** (A) VEN graph demonstrates the number of conserved transcription factor binding sites (TFBS) in the homeologs of *TaD27* gene. (B) Bar chart shows the total number of TFBSs in the homeolog of *TaD27* gene. (C) Regulation of *TaD27* homeologs controlled by distinct transcription factor families. By employing opensource Web tool PlantPAN 2.0 at <http://plantpan2.itps.ncku.edu.tw/promoter.php>, TFBS were forecasted. Graphical illustration was created by using online source RAWGraphs (<https://www.rawgraphs.io/>).
